# Complete genome sequence of an oryctes rhinoceros nudivirus isolated from Korean rhinoceros beetles (*Trypoxylus dichotomus*) in Korea

**DOI:** 10.1016/j.virusres.2023.199167

**Published:** 2023-08-23

**Authors:** Eunsun Kim, Ji-Young Kim, Wontae Kim, Seokhyun Lee, Kwan-Ho Park, June-Sun Yoon

**Affiliations:** a Industrial Insect and Sericulture Division, National Institute of Agricultural Sciences, RDA, Wanju 55365, Republic of Korea; b Department of Agricultural Convergence Technology, Jeonbuk National University, Jeonju 54596, Republic of Korea; c Research Policy Planning Division, RDA, Jeonju, 54875, Republic of Korea; d Department of Animal Biotechnology, Jeonbuk National University, Jeonju 54596, Republic of Korea

**Keywords:** Trypoxylus dichotomus nudivirus (TdNV) genome, Oryctes rhinoceros nudivirus (OrNV), Virus transmission, Genome analysis

## Abstract

Nudivirus-infected Korean rhinoceros beetles (*Trypoxylus dichotomus*) were first identified in 2015, and while a complete genome sequence of the virus has long been uploaded to the NCBI database, it has not been examined in detail. Here, we describe the genomic characteristics of Trypoxylus dichotomus nudivirus (TdNV), which represents a new Oryctes rhinoceros nudivirus (OrNV) strain, isolated from infected *T. dichotomus* in the Republic of Korea. We examined factors derived by the cross-species infection of OrNV from nucleotide levels to the whole genome level. Our genomic analysis study suggests that TdNV-Korea is highly conserved with other OrNVs in terms of genomic structures and genome size. Our investigation of the genomic structure revealed that TdNV-Korea has the least number of open reading frames (ORFs) of all available OrNV genomes; three hypothetical genes were notably absent only in TdNV-Korea. In addition, the genomic alteration of the nudivirus core genes discloses that various amino acid mutations caused by single-nucleotide polymorphism and short indels (insertion/deletion) were found in most of the nudivirus core genes of TdNV-Korea. Our findings provide a valuable resource for those seeking a greater understanding of cross-species nudivirus transmission and will certainly provide valuable insight for reconstruction and reinterpretation of future and previously identified OrNV strains.

## 1 Introduction

The Oryctes rhinoceros nudivirus (OrNV, genus *Alphanudivirus*, family *Nudiviridae*) has long been used to control the population of coconut rhinoceros beetle, which is a serious pest of the coconut palm oil industry in many countries (Huger, 2005; Ramle et al., 2005). As its name represent, this virus's natural host is *Oryctes rhinoceros*. However, it can also affect a variety of other Dynastinae species, including *Scapanes australis, O. boas, O. monoceros, O. nasicornis*, and others with various level of pathogenicity (Gopal et al., 2001). Since the discovery of the virus and subsequent use in the management of Oryctes beetles, it has been introduced into a number of other locations in the Indo-Pacific region. In addition, this virus spread to the Republic of Korea and infected the rhinoceros beetle, *Trypoxylus dichotomus* (Lee et al., 2015).

*T. dichotomus* infected by OrNV was first identified as a major pathogen in the Republic of Korea in 2015 (Lee et al., 2015)*.* In that study, several primer sets for virus diagnosis were designed and the peroral inoculation rate was checked, noting that over 60% of infected larvae were killed within six weeks. The same group also reported that serious structural alterations in the fat body and intestine of *T. dichotomus* were caused by OrNV infection (Kwon et al., 2015). While this group uploaded the raw genome sequences of a nudivirus isolated from *T. dichotomus*, to date no analysis has been published.

Since Wang et al. (2011) posted a complete genome sequence of OrNV from a Malaysia sample (OrNV-Ma07, containing 139 predicted protein-coding open reading frames (ORFs/gps) in 127,615bp) on the NCBI database, genome sequences of OrNV isolated from the Solomon Islands, Indonesia, Philippines, and Palau also have been uploaded. The complete genome sequences of OrNV from the Solomon Islands showed characteristics distinct from those of the OrNV-Ma07 strain, including a 1,698bp shorter genome size and putatively inverted regions around *gp130s* (Etebari et al., 2020a). A complete genome sequence of Oryctes rhinoceros nudivirus strain from Riau Province, Indonesia (named OrNV-LiboV) contains 124,926bp with 123 ORFs (Kurnia et al., 2021). Later, two OrNV strains from Palawan, Philippines (OrNV-X2B) and Melekeok, Palau (OrNV-Palau1) were isolated, and the genome sizes were determined as 125,905bp and 126,039bp, respectively (Tanaka et al., 2021). Recently, two more sequences from Johor, Malaysia were added to the NCBI database; the Batu Pahat and the Kluang strain (Anggraini et al., 2023). Based on various sequencing and assembly techniques, each strain has variable sizes of the genome and anticipated ORF/gps counts. A raw genome OrNV sequence from a Korean sample was deposited in the Sequence Read Archive (SRA) (accession no. SRS2584474), however, no such ORF/gp data or similarity with OrNV strains were demonstrated.

Here, to identify the factors implicated in cross-species transmission of OrNV the SRA data mentioned above were assembled and ORFs/gps were annotated using the raw Trypoxylus dichotomus nudivirus sequencing data isolated from *T. dichotomus* collected in the Republic of Korea (Accession number: PRJNA413966). Hereafter, this virus is referred to as *T. dichotomus* nudivirus-Korea (TdNV-Korea). The genome of TdNV-Korea was compared to that of other OrNV strains in various ways: the size of the genome, annotated ORFs/gps, sequencing methods, and genome regions showing the most differences. To rule out the possibility of sequencing and annotation errors, Sanger sequencing was performed to verify the missing ORF/gp sites from the TdNV-Korea. Then, to identify genetic alterations that may have occurred during the cross-species transmission, a comprehensive analysis of core nudivirus genes (e.g., encoding for factors in transcription, infection, virus packaging, assembly, or morphogenesis of the nudivirus) was performed at the protein level. Our findings provide a valuable resource for those seeking a greater understanding of nudivirus genome analysis and cross-species nudivirus transmission.

## 2 Material and method

### 2.1 Genome Sequence

The raw sequence data were collected from the “OrNV infected Oryctes rhinoceros” entry uploaded by the National Institute of Agricultural Science in Korea (SRA no. SRS2584474 SRR6161627). These raw data were generated by an Illumina HiSeq2500 containing 11.3M spots, 2.3G bases. The raw reads from the SRA database were filtered using FASTP software to remove low-quality reads, which were base-called with an error rate higher than 0.1% (Q-score < 30). The quality-filtered clean reads were subjected to SPAdes assembler v3.14 software to construct a circular form of the viral genome sequence (Antipov et al., 2020). The genome sequence was carefully rechecked by eye and any errors were fixed as necessary. Ultimately, the total sequence of TdNV-Korea was determined to be 126,408bp. The TdNV-Korea genome sequence was predicted to be protein-encoding by both fgenesV0 (http://www.softberry.com/berry.phtml?topic=virus0&group=help&subgroup=gfindv, accessed on 15 March 2023) and ORF Finder (https://www.ncbi.nlm.nih.gov/orffinder/, accessed on 15 March 2023), with the option of methionine-initiated ORFs encoding more than 30 amino acids. For confirmation, each OrNV-Ma07 ORFs was local-blasted into the TdNV-Korea genome using BioEdit. Using NCBI's BLASTP, PSI-BLAST, and TBLASTN tools, sequence similarity comparisons of all predicted ORFs were carried out against public databases. TdNV-Korea ORFs were annotated based on OrNV-Ma07 and we named our ORFs as *TdNV_gp000s*. The Supplementary data contains gene IDs, annotations, strand information, frame information, the start and the end sites, as well as nucleotide and amino acid sequences of all genes, and percent identity compared to OrNV-Ma07 amino acid sequences (Sup. Data 1).

### 2.2 *In vitro* confirmation of missing ORFs/gps

Once assembly and annotation were completed, ORFs/gps were compared to the OrNV reference genome, OrNV-Ma07. Eighteen ORFs/gps were different from the OrNV-Ma07 strain, in terms of missing or fused ORFs/gps. To rule out the possibility of sequencing errors that may come from the Illumina sequencing, primers were designed to amplify the 18 ORFs/gps (Sup. Table 1). Then, sequences were obtained by Sanger sequencing (Bioneer, Korea). *T. dichotomus* DNA was extracted using Clear-S Quick DNA extraction kit (IVT3002, Invirustech Co., Korea) following the manufacturer's protocol. PCR amplifications were done in 20 μl reactions with 5 mM forward and reverse primers, 10 μl of 2X AccuPower Taq PCR Master Mix (K-2609, Bioneer, Daejeon, Korea) and 20 ng of template. PCR conditions were 95 ˚C for 1 min, followed by 35 cycles of 95 ˚C for 30 s, 55 ˚C for 30 s and 72 ˚C for 1 min, finishing with an extension step at 72 ˚C for 5 min. PCR products were purified using the Clear-S PCR/Gel DNA fragment purification kit (IVT3005, Invirustech Co., Korea). Then, the samples were sequenced by the Sanger sequencing method (Bioneer, Daejeon, Korea). The sequencing data were examined to confirm whether the previous Illumina genomic sequencing data were identical to the Sanger sequencing data using BioEdit for local blast, Clustal Omega for alignments Omega (https://www.ebi.ac.uk/Tools/msa/clustalo/, accessed on 15 March 2023), several blast options, and the ExPASy translation program (https://web.expasy.org/translate/, accessed on 15 March 2023).

### 2.3 Genomic alterations in core genes

The 28 nudivirus core genes, coding for transcription, infection, virus packaging/assembly/ morphogenesis and DNA replication/repair/recombination factors, were collected from five known OrNV strains in the NCBI database (Etebari et al., 2020b; Petersen et al., 2022). OrNV-Batu Pahat and Kluang data were omitted due to their incomplete annotation. The protein sequences of each nudivirus core gene were aligned with Clustal Omega. The size of the protein, type of mismatches, mismatch positions, and domain sites in TdNV-Korea were compared to other OrNV strains. Twenty-eight core genes were collected and comparison data were Supplementary data 2.

### 2.4 Estimates of evolutionary divergence between sequences

Kimura 2-parameter analyses were conducted using the Kimura 2-parameter model (Kimura, 1980). The number of base differences per site between sequences was analyzed using the p-element model (Table 4).

## 3 Results

### 3.1 Genetic diversity of TdNV

Information regarding the complete OrNV genome sequences were collected from the NCBI database: Malaysia (OrNV-Ma07), the Solomon Islands (OrNV-SI), Indonesia (OrNV-LiboV), Philippines (OrNV-X2B), Palau (OrNV-Palau1), and Malaysia (OrNV-Batu Pahat and Kluang). When we compare the sizes of the genomes, the genome of TdNV-Korea is 126,408bp, which is 1,207bp shorter than OrNV-Ma07 but 491bp more than the OrNV-SI strain (Table 1). As for the number of ORFs, TdNV-Korea has the least within OrNVs (Table 1). Depending on the advances in sequencing technology of that time, different sequencing methods and versions were used. Moreover, various assembly methods were used by different groups. A list of annotated ORFs/gps of TdNV is provided in Supplementary data 1. including its Gene ID, annotation, strand information, frame information, the start and end nucleotide sites, length of CDS (coding sequence), amino acid number, and nucleotide sequences (Sup. data 1).

**Table 1:** Complete genome sequences of Oryctes rhinoceros nudivirus and Trypoxylus dichotomus nudivirus from NCBI database

Isolate	Origin	GenBank ID	Size, bp	Annotated ORFs	Sequencing Methods	Assembly Method	Annotation method	Refs.
OrNV-Ma07	Malaysia	EU747721	127,615	139	Illumina NovaSeq 6000	MetaviralSPAdes	ORF Finder, GeneQuest & BioEdit	Wang et al. (2011)
OrNV-Solomon Islands	Solomon Islands	MN623374	125,917	130	Oxford Nanopore Technologies	Flye v. v.2.5	Prokka	Etebari et al. (2020a)
OrNV-LiboV	Riau, Indonesia	MZ727584	124,926	123	Illumina NovaSeq 6000	megahit v. 1.2.9; lastz v. 1.04.00	Geneious 2019.1.1	Kurnia et al. 2021)
OrNV-X2B	Palawan, Philippines	MW298153	125,905	132	Illumina HiSeq 2500	unicycler v. 0.4.8; minimap2 v. 2.17; bcftools v. 1.10.2	fgenesV0, Vgas	Tanaka et al. (2021)
OrNV-Palau1	Melekeok, Palau	MW298154	126,039	129	Illumina HiSeq 2500	unicycler v. 0.4.8; minimap2 v. 2.17; bcftools v. 1.10.2	fgenesV0, Vgas	Tanaka et al. (2021)
OrNV- Batu Pahat	Johor, Malaysia	ON931348	124,925	126	Illumina NovaSeq 6000	MetaSPAdes v. 3.9.0	Prokka	Anggraini et al. (2023)
OrNV-Kluang	Johor, Malaysia	ON931347	125,794	125	Illumina NovaSeq 6000	MetaSPAdes v. 3.9.0	Prokka	Anggraini et al. (2023)
TdNV-Korea	The Republic of Korea	BK063656	126,408	121	Illumina HiSeq2500	SPAdes v3.14	ORF Finder & BioEdit	this study

**Fig. 1:**
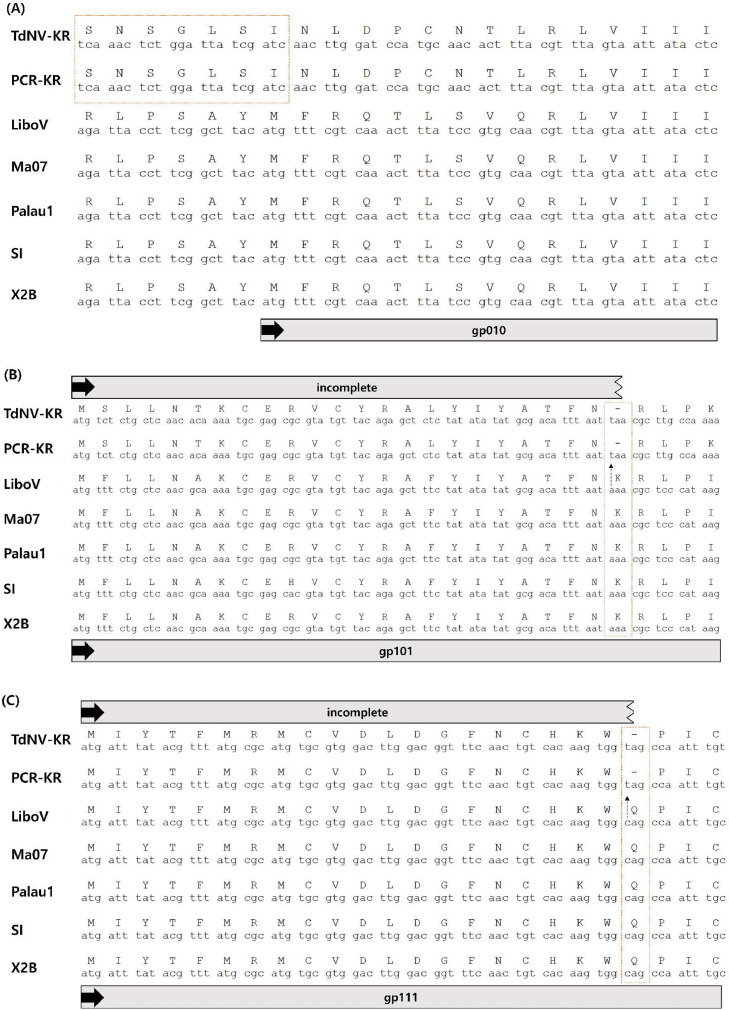
The alignment of three absent ORFs/gps in TdNV (A) *gp010*: a start codon was not found at the beginning of *gp010*. (B) *gp101*: an SNP (AAA → TAA) created a stop codon in the middle of *gp101*. (C) *gp111*: an SNP (CAG → TAG) created a stop codon in the middle of *gp111*. TdNV-KR: TdNV-Korea; PCR-KR: Sanger sequencing result of TdNV-Korea; LiboV: OrNV-LiboV; Ma07: OrNV-Ma07; Palau1: OrNV-Palau; SI: OrNV-Solomon Islands; and X2B: OrNV-X2B. Bold arrows indicate the start codon (ATG). Dotted arrow indicates the SNP.

Once assembly and annotation were done, we found 18 ORFs/gps were absent or fused from TdNV-Korea genome compared to OrNV-Ma07 strain (Table 2). To verify the absence or fused form of ORFs/gps and rule out the possibility of sequencing errors, primers were designed for Sanger sequencing targeting each ORFs/gps. The Sanger sequencing results were noted as “PCR-KR” in Fig. 1. Out of 18 absences and fusions, three ORFs/gps were missing only in TdNV-Korea. The absence of *gp010* derived from the lack of a start codon; not specifically derived from a SNP of a start codon, but no such start codon sequences was found at the beginning of the sequence (Fig. 1 (A)). In the case of *gp101*, a AAA sequence became TAA which created a stop codon in the middle of the sequence (Fig. 1 (B)). The sequence of *gp111* also generated an incomplete ORF/gp, due to an SNP producing a stop codon in the middle of the sequence, CAG to TAG (Fig. 1 (C)). These examples of genetic diversity could impact on virus transmissibility from species to species and continents to continents.

**Table 2:** Genes not present in TdNV-Korea

OrNV-Ma07 gp Number	Gene Annotation	TdNV-Korea	OrNV-Ma07	OrNV-Solomon Islands	OrNV-Palau1	OrNV-LiboV	OrNV-X2B
*gp010*	hypothetical protein	**XX**	O	O	O	O	O
*gp032*	hypothetical protein	X	O	X	O	X	X
*gp049*	hypothetical protein	X	O	O	X	X	O
*gp050*	hypothetical protein	X	O	X	O	X	X
*gp066*	hypothetical protein	X	O	X	O	X	O
*gp068*	hypothetical protein	X	O	X	O	X	O
*gp070*	hypothetical protein	X	O	O	X	X	X
*gp077/gp078*	semaphorin protein	**F**	O	O	O	**F**	O
*gp081*	hypothetical protein	X	O	X	O	X	O
*gp082*	hypothetical protein	X	O	X	X	X	X
*gp084/gp083*	polysaccharide lyase family 6-like protein	**F**	O	O	**F**	**F**	**F**
*gp085*	hypothetical protein	X	O	O	X	O	X
*gp091*	hypothetical protein	X	O	X	X	X	X
*gp099*	Ac120-like protein	X	O	O	X	O	O
*gp101*	hypothetical protein	**XX**	O	O	O	O	O
*gp111*	hypothetical protein	**XX**	O	O	O	O	O
*gp129*	hypothetical protein	X	O	X	X	X	X
*gp130*	hypothetical protein	X	O	X	O	X	O

O: presence; X: absence; **F**: Fused; **XX**: only absence in TdNV.

**Fig. 2:**
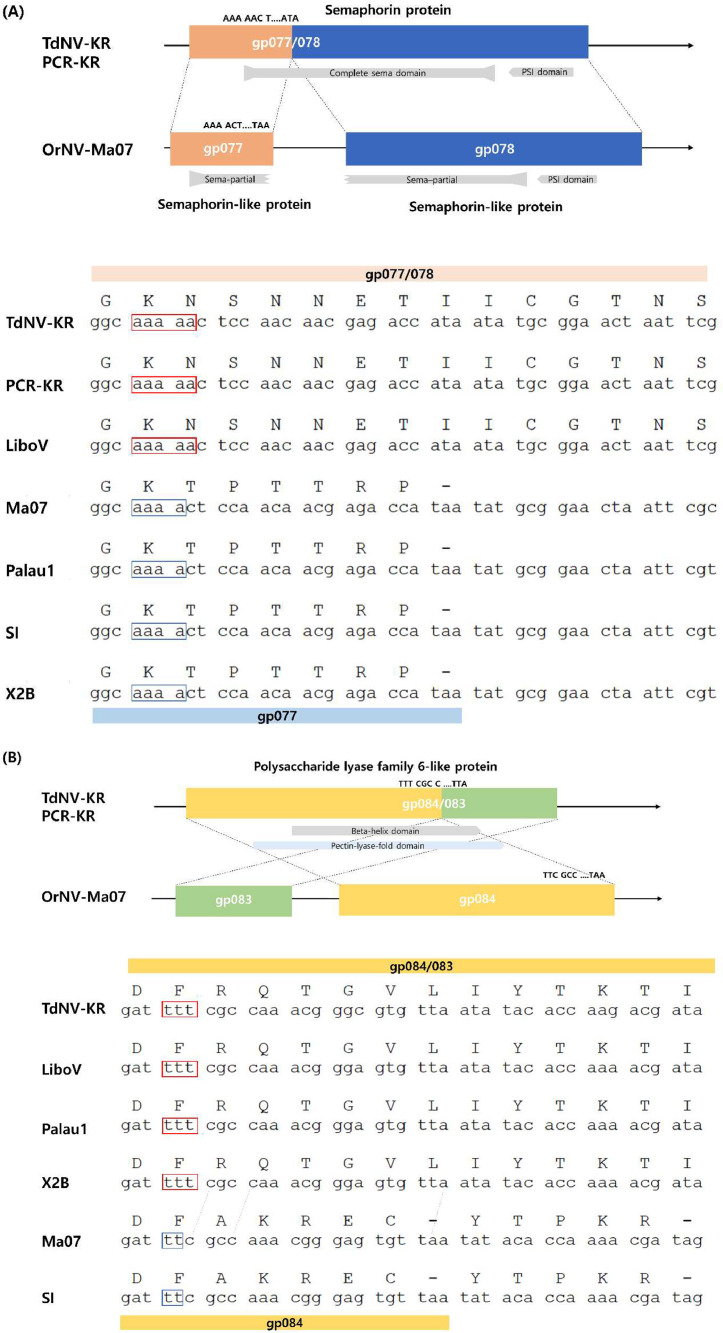
Characterization of fused ORFs/gps (A) *gp077/078*: Insertion of an A nucleotide induces the frameshift that forms the complete Sema domain. (B) *gp084/083*: Insertion of a T nucleotide induces the frameshift that forms the complete beta-helix domain. TdNV-KR: TdNV-Korea; PCR-KR: Sanger sequencing result of TdNV-Korea; LiboV: OrNV-LiboV; Ma07: OrNV-Ma07; Palau1: OrNV-Palau; SI: OrNV-Solomon Islands; and X2B: OrNV-X2B.

**Fig. 3:**
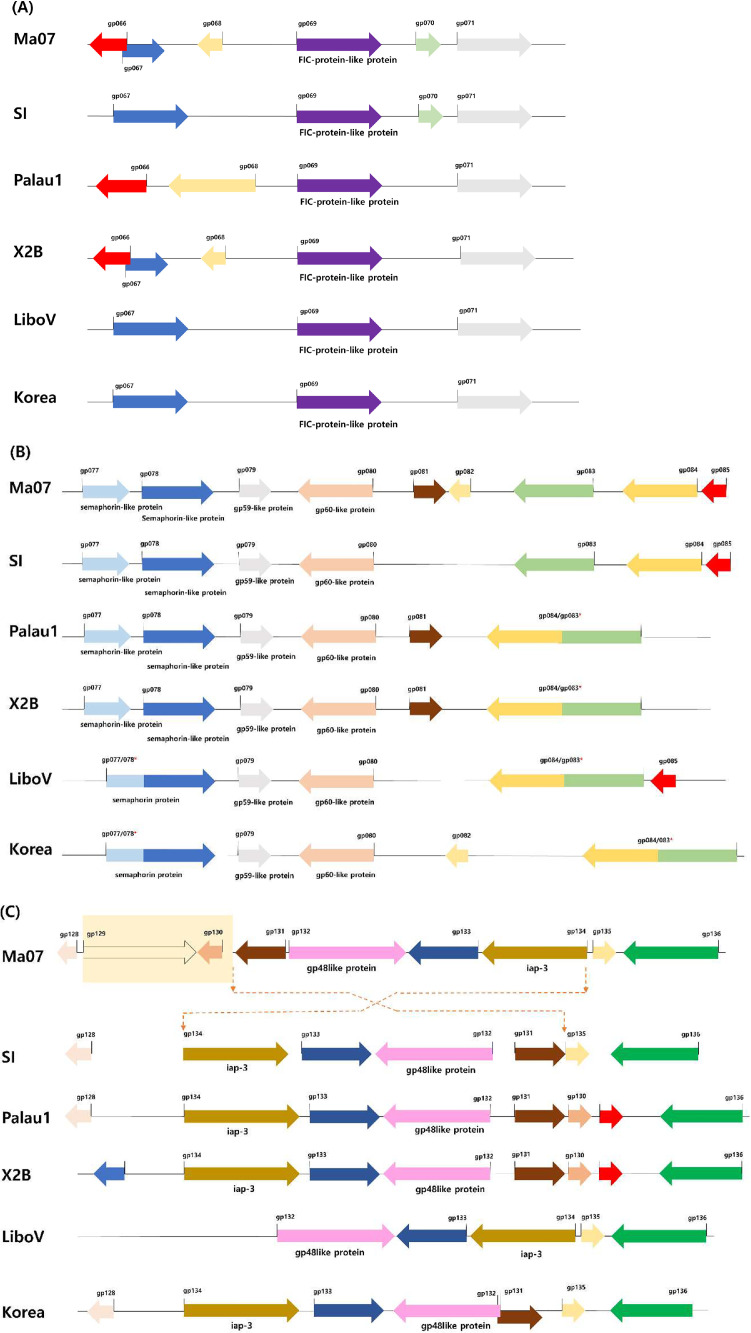
Genome alignment of the three controversial regions: (A) Genomic variability between *gp066* to *gp071*. (B) Genomic variability between *gp077* to *gp085*. (C) Genomic variability between *gp128* to *gp136*. If gp/ORF is annotated, the annotated name is inserted under the arrow. The arrow indicates the direction of gp/ORF on the genome. TdNV-KR: TdNV-Korea; LiboV: OrNV-LiboV; Ma07: OrNV-Ma07; Palau1: OrNV-Palau; SI: OrNV-Solomon Islands; and X2B: OrNV-X2B.

The ORFs of *gp077* and *gp078* are known to be present separately in most of the OrNV strains. except in the OrNV-LiboV annotations. Both *gp077* and *gp078* encode for semaphorin-like proteins and are combined in the cases of TdNV-Korea strain and OrNV-LiboV, due to insertion of an A nucleotide, which causes a frameshift in *gp077* and a fusion with *gp078* (Fig. 2). This combined ORFs/gps leads to the incomplete semaphorin-like proteins being joined into the complete semaphorin protein, containing an approximately 445 amino acid semaphorin domain and a plexin-semaphorin-integrin (PSI) domain. According to the OrNV-LiboV annotation, *gp077* seems to be missing in the NCBI database. However, once we investigated this at the nucleotide level, we found the insertion of an A nucleotide at the end of OrNV-LiboV *gp077*, just as in TdNV-Korea (Figs. 2 & 3), suggesting that lack of further investigation after the annotation limits the genome analysis.

*Gp083* and *gp084* showed not only a fused but also reversed order. Out of five known OrNV strains, three strains contained *gp084/083*, whereas two strains contained completely separate *gp083* and *gp084*. These were the reference genome, OrNV-Ma07, and the long-read sequencing genome, OrNV-SI (Fig. 2). Similar to *gp077/078*, an insertion of a T nucleotide lead to the frameshift which skips the termination codon and continues as the beta helix domain in between gps (Fig. 2). Due to the merging, *gp084/083* became a functional gene, polysaccharide lyase family 6-like protein containing right handed beta helix regions.

We found three regions of the genome displaying the distinct characteristics of TdNV-Korea vs. other OrNV strains (Fig. 3). One significant variation in the ORFs/gps number occurs in the region between *gp066* and *gp071* (Fig. 3 (A)). Within this small region, three out of six genes were absent in TdNV-Korea (*gp066, gp068*, and *gp070*) compared to OrNV-Ma07. OrNV-SI lost *gp066* and *gp068*, and OrNV-Palau1 lost *gp67* and *gp70*. All those genes showing alterations in their presence were annotated as hypothetical proteins (Fig. 3 (A)).

According to a previous report by (Tanaka et al., 2021), OrNV-X2B (MW298153) and OrNV-Palau1 (MW298154) contained 132 and 129 annotated ORFs (Table 1). Searching through the NCBI database, however, one more ORF from each isolate were found, making the total 133 and 130 ORFs/gps, respectively. These differences come from the merger of *gp083* and *gp084*. It was challenging to determine whether *gp083* was distinctly present in the genomes or combined with *gp084* (Fig. 3 (B)). This is because the order of *gp083* and *gp084* are simultaneously inverted and merged, as can be seen in the OrNV-Palau1, OrNV-LiboV, OrNV-X2B, and TdNV-Korea isolates. For this reason, we designated this ORF/gp as *gp084/083* (Fig. 3 (B)). In addition, we found another merged region, *gp077/078* that OrNV-LiboV and TdNV-Korea have similar unusual features (Fig. 3 (B)).

Concerning *gp128* to *gp137* region, TdNV-Korea was highly conserved with the OrNV-SI strain pattern (Fig. 3 (C)). First, *gp130* to *gp135* regions were inverted (relative to the OrNV-Ma07), just as in the OrNV-SI, X2B, and, Palau1 strain sequences. Second, *gp129* and *gp130* were omitted in TdNV-Korea, as well.

### 3.2 Genomic alterations in core genes

Virus transmissibility could be affected by the genomic alterations in core genes known to be involved in transcription, infection, virus packaging, assembly, morphogenesis and so on. Here, we used multiple sequence alignment to align 28 nudivirus core genes of TdNV-Korea and other OrNVs, at the protein level, to check the nonsynonymous mutations, insertions and deletions, and investigate whether those mutations were present in any domain sites (Table 3& Sup. Data 2).

**Table 3:** Genomic alterations in nudivirus core genes of TdNV compared to OrNVs

Functions	Gene	Size of the Protein	TdNV mismatches	Deletion	Insertion	Domain sites
Transcription	*lef-3*	172	3			
	*lef-4*	401	8			
	*lef-5*	78	0			
	*lef-8*	922	8			
	*lef-9*	495	73	61		
	*p47*	324	2			
Infectivity	*pif-0/p74*	736	13			5
	*pif-1*	491	7			
	*pif-2*	374	4			4
	*pif-3*	204	1			1
	*pif-4/19kDa*	255	2			
	*pif-5/odv-e56*	411	8			
	*pif-6/ac68*	136	0			
Packaging, assembly, morphogenesis	*pif-8/vp91*	659	16			
	*38K*	279	2			
	*p33/ac92*	424	12			
	*vlf-1*	703	18	2	1	8
	*vp39*	250	2			
	*ac81*	158	9	7		1
DNA replication, repair, recombination	*dnapol*	1281	26	1	2	11
	*helicase*	1240	12			
	*helicase-2*	821	15		1	
	*integrase/rec*	364	5			3
Unknown function	*GbNV gp19-like*	278	5			
	*GbNV gp51-like*	110	7	4		
	*GbNV gp58-like*	51	1			
	*GbNV gp67-like*	368	20	2	1	
	*GbNV gp72-like*	248	12		5	

Among those genes involved in transcriptions, such as *lef-3, lef-4, lef-5, lef-8, lef-9*, and *p47*, no mismatches were found only in *lef-5*. On the other hand, TdNV-Korea *lef-*9 has 73 different amino acid alterations compared to other OrNV strains due to the different start codon prediction (Table 3).

Within the core genes encoding for virus infectivity, such as *pif-0/p74, pif-1, pif-2, pif-3, pif-4/19kDa, pif-5/odv-e56* and *pif-6/ac68*, we detected the most amino acid changes in *pif-0/p74*: 13 amino acids alterations (Table 3). Among these 13 mismatches, five were in the domain sites: two in the Baculo_p74 domain site and three in the Baculo_p47 N domain site. The *pif-5/odv-e56* showed eight amino acids alterations and *pif-1* contained seven amino acids alterations in TdNV compared to OrNVs. There were no such modifications found in *pif-6/ac68*.

Those genes involved in packaging, assembly, morphogenesis were *vp91/pif-8, 38K, p33/ac92, vlf-1, vp39,* and *ac81,* capsid proteins (Table 3). The *vlf-1* showed 18 mismatches containing two deletion parts and one insertion site and 15 nonsynonymous mutations. When examining domain searches, eight out of 18 mismatches were belonged to the SMC (structural maintenance of chromosomes) domain site (Sup. Fig. 1).

We also investigated the genes responsible for DNA replication, repair, and recombination such as DNA polymerase, helicase, helicase-2, and integrase, at the protein level. We found the greatest number of mismatches in the DNA polymerase gene. In this gene, two out of 26 mismatches were in the polymerase B domain site and nine of them were in the polymerase 2 domain sites (Table 3). Moreover, one deletion and two insertions of amino acid mutations was observed (Table 3).

### 3.3 Estimating genetic differences between six whole virus genome sequences

Eight whole genome sequences were collected from the NCBI database including TdNV-Korea. OrNV-Batu Pahat and Kluang data were omitted due to their incomplete annotation. A pairwise comparison analysis was performed between five OrNVs and the TdNV-Korea samples using the p-distance (bottom left) and Kimura 2-parameter (top right) method (Table 4). The p-distance result suggests that TdNV-Korea is relatively less related to OrNV-Ma07 and OrNV-LiboV (94.53% and 95.17% respectively), and more closely related to other samples (all above 96% identity). An identity of greater than 99.99% was found between OrNV-SI and OrNV-X2B. Results from the Kimura 2-parameter and p-distance tests were substantially conserved overall.

**Table 4:** Genetic distance between the six known whole genome sequences of the OrNVs and TdNV

	TdNV-Korea	OrNV-Ma07	OrNV-LiboV	OrNV-X2B	OrNV-Solomon Islands	OrNV-Palau1
TdNV-Korea		0.05717	0.05017	0.03779	0.03757	0.0387
OrNV-Ma07	0.05478		0.00111	0.02359	0.02311	0.02405
OrNV-LiboV	0.04830	0.00111		0.01821	0.01786	0.01893
OrNV-X2B	0.03668	0.02321	0.01798		0.00107	0.00317
OrNV-Solomon Islands	0.03647	0.02274	0.01764	0.00107		0.00284
OrNV-Palau1	0.03754	0.02365	0.01868	0.00316	0.00284	

Genetic distance was calculated by p-distance (bottom left) and kimura 2-parameter (top right) method using Mega11 software.

## 4 Discussion

Since TdNV-Korea was first reported by the Korean Rural Development Administration in 2015, the entire genome sequence of TdNV-Korea was eventually uploaded to the NCBI in 2017. In this study, we report on the genomic characterization of *T. dichotomus* nudivirus, TdNV-Korea, as infected in *T. dichotomus* isolated from the Republic of Korea, and highlight consequences of genetic diversity caused by the cross-species infection of OrNV.

Sequencing quality can vary depending on the sequencing methods, due to the short-read and the long-read sequencing technology and the version of the technology that developed at that time. The long-read sequencing technology tends to eliminate amplification bias and generate a reasonable length to overlap a sequence for better sequence assembly (Amarasinghe et al., 2020; Sevim et al., 2019). Thus, long-read sequencing provides more comprehensive information about the genome structure and can be valuable for *de novo* genome assembly, resolving structural variations, etc. (Amarasinghe et al., 2020; Sevim et al., 2019). The drawback is that, compared to the short-read sequencing, the accuracy per read may be much lower. On the other hands, the short-read sequencing technology has high depth and high-quality data for the lowest cost per base. The size of the genome predicted varies depending on the assembly programs (Sevim et al., 2019). In addition, ORF prediction could come out differently based on assembly methods; the virome characterization is critically impacted by the choice of assembly software (Sutton et al., 2019). Therefore, to get the most accurate data, combining short and long read data can be advantageous. In our study based on Illumina sequencing and SPAdes assembler, we were note able to find 18 ORFs compared to the reference genome, OrNV-Ma07, indicating that annotation errors could come from the uncertainly of sequencing and assembly errors. To verify the existence of 18 ORFs, we used the Sanger sequencing method, by designing primers to sequence each ORF. For example, when we targeted for the *TdNV_gp010*, we designed a forward primer at the end of the *TdNV_gp009* and a reverse primer at the beginning of the *TdNV*_*gp011* so that we were able to see the whole sequence of *TdNV_gp010*. Overall, the Sanger sequencing results were highly conserved with the given genome sequences, sufficient to make decision about SNPs and indels (insertion/deletion) in 18 ORFs/gps, indicating that the procedures for assembly and annotation were quite accurate in our study. This type of genomic alteration could be due the evolutionary changes or regional changes which contribute to the adaption to the new host or new environment. It will be challenging to get correct interpretation if you do not thoroughly analyze the genome ORF by ORF.

In the case of *TdNV_gp010*, an intergenic region within the viral genome was completely different to that of other OrNVs, in that no start codon was found (Fig. 2(A)). In the case of *TdNV_gp101*, a stop codon generated an incomplete ORF/gp in the SNP that codes for lysine (AAA) into one that codes for the stop codon (TAA) (Fig. 2(A)). Similar to the TdNV_gp101 mutation, glutamine (CAG) within the sequence of *TdNV_gp111* was altered to a stop codon (TAG) (Fig. 2(A)). As all those missing gps were hypothetical proteins, it was challenging to understand the entire implications of the absences. However, those absence in TdNV could be the factors for contributing to the adaption to the new host or new environment.

In case of a fusion site in *TdNV_gp077/078*, insertion of an A at the end part of the *TdNV_gp077* sequence led to the frameshift, which caused *TdNV_gp077* to continue into *TdNV_gp078*. This phenomenon was observed in OrNV-LiboV strain as well: *gp077* was annotated as “misc. feature (a miscellaneous feature)” in the NCBI database and *gp078* was solely annotated. Once we investigated the whole genome sequence of OrNV-LiboV in the nucleotide level, however, OrNV-LiboV strain showed the insertion of an A at the end part of *gp077* sequence similar to that of TdNV (Fig. 2). In OrNV-Ma07 and SI the sequence indicated both *gp077* and *gp078* were semaphorin-like proteins due the existence of the Sema (semaphorin) domain, a protein interacting module, of semaphorin 1A. However, when we looked closely into the domain structure, *OrNV-gp077* had the partial front of the Sema domain, of around 70 amino acids, and *OrNV-gp078* had the remaining domain sites, of about 350 amino acids, followed by a conserved PSI domain, a cysteine repeat. By contrast, TdNV-Korea and OrNV-LiboV contained the entire Sema domain, of about 445 amino acids, which could be the complete domain (Fig. 2(A)) followed by the PSI domain. This *gp077/078* fused gene, containing the Sema domain and the plexin repeat domain, forms the fully functional structure of the semaphorin protein (Fig. 2(A)). The semaphorin protein is known to play a role in the development of the nervous system and in axonal guidance (Yazdani and Terman, 2006). Functions of semaphorin in other viruses. such as Singapore grouper iridovirus-encoded semaphorin homologue, were in viral replication, cytoskeleton reorganization, and inhibition of cellular immune responses (Yan et al., 2014). The fusion we mentioned above is not uncommon in virus genomes. Fusions of adjacent ORFs were founds in four different regions in AcMNPV-C6 against the AcMNPV-WP10 consensus sequence (Chateigner et al., 2015). However, it is not common that two ORFs are both reversed in order and fused such as *gp084/083* (Figure 1(B)). These kinds of fusions were found not only in TdNV-Korea, but also in OrNV-Palau, LiboV, and X2B strains. This kind of genetic alterations make it difficult to justify the number of ORFs in the OrNV genome analyses. These fusion ORFs/gps showed the common phenomenon that a domain is completed by fusion of two ORFs/gps, resulting in a single complete gene (Figure 2(B)). In this case, the combination of *gp084/083* becomes a polysaccharide/pectin lyase family 6-like protein, according to NCBI blast results. When we examined the domain and its structures by the InterProScan search, these combined ORFs/gps contain a polysaccharide/pectin lyase fold which acts as virulence factors in *Aspergillus* species (Mayans et al., 1997). This suggests that expression of specific genes by SNPs and indels or fusions of ORFs/gps could chronically affect the virulence of the virus and virus transmissibility by interacting with immune response.

There are 32 core genes assigned to all nudivirus species divided into functional categories such as those genes involved in transcription, infectivity, packaging/assembly/morphogenesis, DNA replication/repair/recombination, nucleotide metabolism and some unknown function genes (Burke, 2019; Cheng et al., 2020; Zhang et al., 2020). In this study, out 28 core genes we found in TdNV-Korea, two genes (*lef-5* and *pif-6/ac68*) were perfectly match with other OrNVs. Considering all the mismatches, mismatches in TdNV-Korea against all OrNV strains showed the greatest number within this core gene list, suggesting that the virus somehow modifies its genome to adapt to the new species and environment. According to a Drosophila innubila Nudivirus study, *vlf-1, pif-1,* and *pif-3* may be crucial for adapting to a new host (Hill and Unckless, 2018). Each gene is highly conserved in its respective group in terms of a gene size and percent identity. There are different types of mutations present, such as SNPs and short indels, the later consisting of two deletion regions, one insertion and 15 mismatches of amino acids in *vlf-1*. Interestingly, eight out of 15 mismatches are within a specific domain site; i.e., the chromosome segregation protein SMC domain (Sup. Fig. 1). In the case of *pif-1*, seven nonsynonymous mutations were found. Only one amino acid was a nonsynonymous mutation in *pif-3*. Nudivirus core genes in TdNV-Korea showed greater genomic variations against OrNV strains than geographical variations within OrNV strains (Etebari et al., 2020b). We believed that potentially only a few of these variations in the genome are involved in functional changes and can potentially alter the characteristic functions while cross-species virus transmission (Parrish et al., 2008).

## 5 Conclusion

It is not sufficient to conclude a genome structure and ORFs map with a single sequencing method and a programmed annotation system. Cutting-edge sequencing technology and multiple sequencing methods with suitable assembly and annotation skills will bring out a trustworthy genome analysis result. Unique features such as fused ORFs or reversed and fused ORFs could be omitted easily, due to either the uncertainly of the sequence or the particular programmed annotation system. There are still vague OrNV-genomes in NCBI database and some of OrNVs are recently updated with poor annotations. To understand the genome of OrNV profoundly, the sequences of more OrNV or TdNV strains, determined using different sequencing technologies, need to be accumulated, while, at the same time, the previously annotated OrNV genomes need to be reinterpreted. Our findings will definitely provide valuable insight for reconstruction and reinterpretation of future and previously identified OrNV strains.

## CRediT authorship contribution statement

**Eunsun Kim:** Conceptualization, Methodology, Investigation, Data curation, Visualization, Writing – original draft. **Ji-Young Kim:** Conceptualization, Methodology, Data curation, Visualization. **Wontae Kim:** Conceptualization, Resources, Methodology. **Seokhyun Lee:** Conceptualization, Resources, Writing – review & editing. **Kwan-Ho Park:** Conceptualization, Investigation. **June-Sun Yoon:** Conceptualization, Data curation, Supervision, Visualization, Writing – review & editing.

## Declaration of Competing Interest

The authors declare that they have no competing financial interests

## Data Availability

Data will be made available on request.

## Appendix Supplementary materials

Image, application 1

Image, application 2

Image, application 3

Image, application 4
