# Supplementary material for: Complete genome sequence of an oryctes rhinoceros nudivirus isolated from Korean rhinoceros beetles (Trypoxylus dichotomus) in Korea
Source: Virus Res. 2023 Aug 23;335:199167. doi: 10.1016/j.virusres.2023.199167 (PMC10485680; doi:10.1016/j.virusres.2023.199167)
Supplement: Supplementary file 3 [file mmc3.docx]

**Supplementary Table 1. Primer List for Sanger sequencing**

| **Target Gene** | **Target Sites: End & Beginning of gp000** | **Forward** | **Reverse** |
| --- | --- | --- | --- |
| **gp010** | **gp009 - gp011** | CAACAGTGTGACTTTGGATGATTTA | ACGATGACATCTCGAGAAACAG |
| **gp032** | **gp031-gp032** | CAGTTGTCCGCCATTACCT | CCCAACGACATCCACTAATCA |
| **gp049-050** | **gp048-gp051** | GGAAACTCAAATCAAAGTAGTACCC | GTGTTGACGAGAGCGAGTAA |
| **gp066** | **gp065-gp067** | GCACCATCAGCGTCTTCTAA | CGCGCATACGTCGTAAACTA |
| **gp068** | **gp067-gp069** | TCGTCACCCATCGTCAATAC | CACACCGAATCACGAACAAC |
| **gp070** | **gp069-gp071** | GATAGTACCACGGTATGGTATGTAAT | ACTATACCGCTGTCTGATTTATCC |
| **gp078** | **gp077- gp078** | GCGGTCGAAACCACATCTA | CGTAGTGGCTGTGTACAGTT |
| **gp081-082** | **gp080-gp083** | GATCACGCAACGCTCTAAGT | ACATGACGCGATCAACAAGA |
| **gp085** | **gp084-gp086** | CACGCACAAATACGCACAAT | AGCGCATATAAGCAAGTCTACA |
| **gp091** | **gp090-gp092** | GGCACACACGACTAACACTA | CAGCGCGTGCAAAGTTC |
| **gp099** | **gp098-gp100** | ACACCCTAGGTGCAATGT | CATATAAACACTCGCTCGCG |
| **gp101** | **gp100–gp102** | CGTGCTAACAATTTAGCGTACC | ATGCGAGCGCGTATGTTA |
| **gp111** | **gp110-gp112** | CGAAACGATGAACTTGGATGTC | GTGTGTGTATGTGTGTGTGTATG |
| **gp129-130** | **gp128-gp134** | GGTTGAAATGTCGAAGGTGTC | TTTCTACTCTTCCGCACAATG |
