# Supplementary material for: Complete genome sequence of an oryctes rhinoceros nudivirus isolated from Korean rhinoceros beetles (Trypoxylus dichotomus) in Korea
Source: Virus Res. 2023 Aug 23;335:199167. doi: 10.1016/j.virusres.2023.199167 (PMC10485680; doi:10.1016/j.virusres.2023.199167)

**Supplementary Figure 1. Different types of mutations present in vlf-1 gene** two deletion regions, one insertion and 15 mismatches of amino acids were observed. Eight out of 15 mismatches are within a SMC-pro domain site, the chromosome segregation protein SMC domain (SMC-prok domain). KR: TdNV-Korea; LiboV: OrNV-LiboV; Ma07: OrNV-Ma07; Palau1: OrNV-Palau; SI: OrNV-Solomon Islands; and X2B: OrNV-X2B.
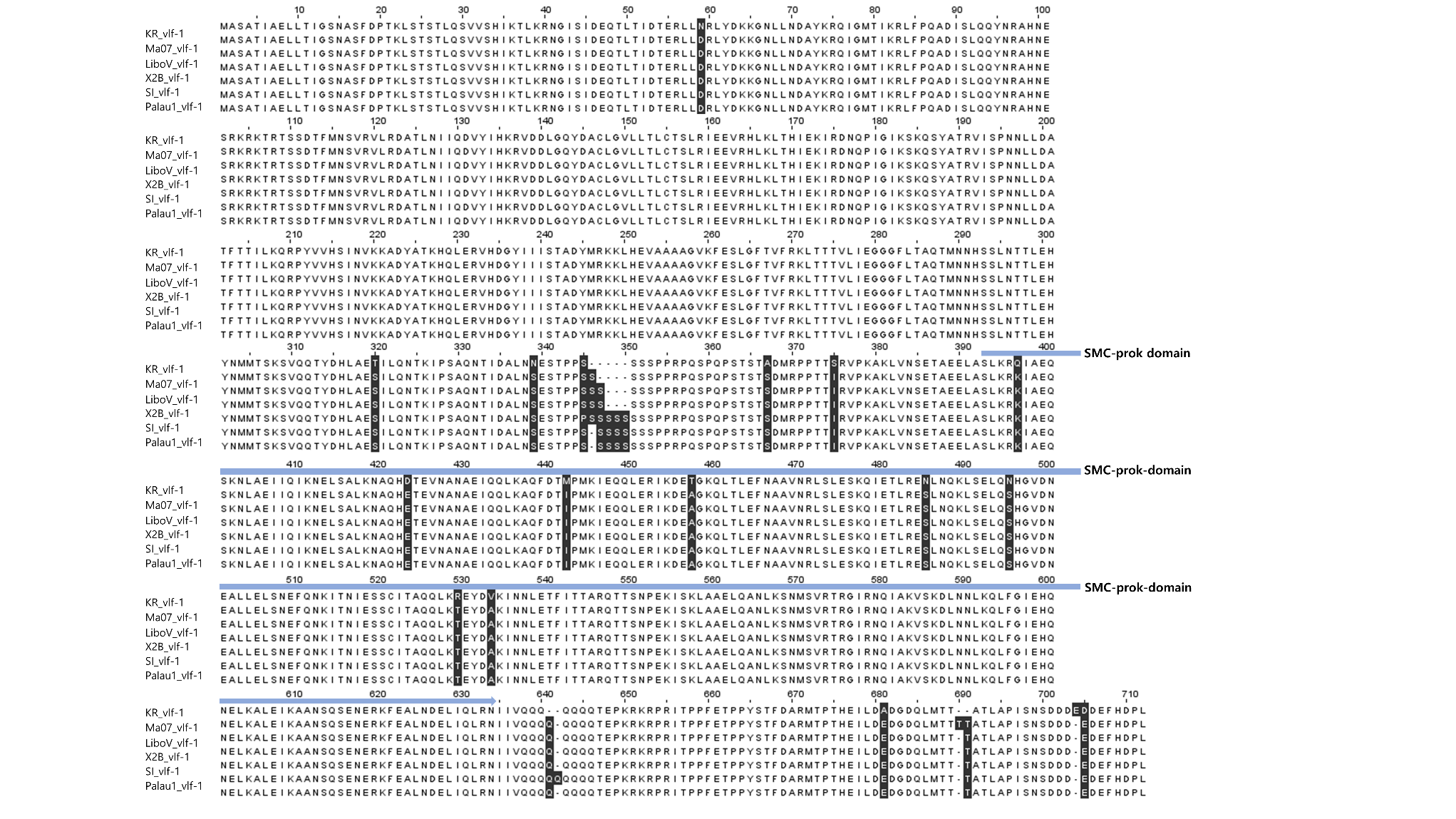

Supplement: Supplementary file 4 [file mmc4.docx]
